# Cardiac transplantation as resolution for Uhl's anomaly: A case report

**DOI:** 10.1016/j.jhlto.2025.100343

**Published:** 2025-07-11

**Authors:** Juan J. Bacigalupe, Natalia Vensentini, Santiago Torroba, Javier A. Mariani, Sandra Defelitto, Norberto Blanco, Fabian Paetz, Julieta Ximena Saenz, Marcelo Nahin, Maximiliano de Abreu

**Affiliations:** a Cardiology Department, Hospital El Cruce, Buenos Aires, Argentina; b Cardiac Surgery Department, Hospital El Cruce, Buenos Aires, Argentina; c Pathology Department, Hospital El Cruce, Buenos Aires, Argentina

**Keywords:** Uhl anomaly, cardiac transplantation, heart failure, Congenital heart disease, Ventricular arrhythmia

## Abstract

Uhl's anomaly is an extremely rare congenital cardiac defect characterized by the absence of myocardium in the right ventricle. It has a poor prognosis, typically fatal during the perinatal period, with very few patients reaching adulthood. We present the case of a 28-year-old woman with heart failure and ventricular arrhythmia associated with Uhl's anomaly, who required cardiac transplantation, with favorable postoperative outcomes. Uhl's anomaly represents an exceedingly rare cause of cardiac transplantation in adults.

## Background

Uhl’s anomaly is a rare congenital heart defect marked by near-complete absence of right ventricular myocardium. Though usually diagnosed in infancy, some patients survive into adulthood. The disease progresses with right heart failure, arrhythmias, and risk of sudden death. Prognosis is poor, with no definitive surgical cure.^1^, ^2^

### Clinical case

**Figure 1:**
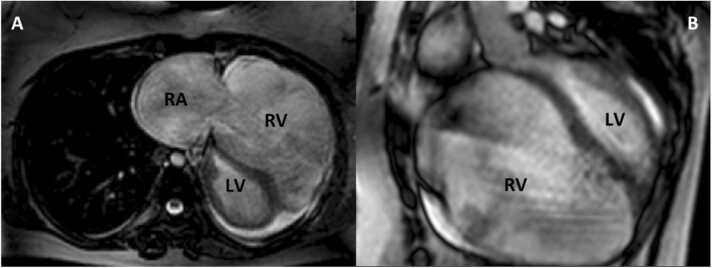
Cardiac magnetic resonance (CMR). (A) Significant dilation of the right atrium (RA) (area: 57 cm²) and right ventricle (RV). (B) Sagittal CMR demonstrating marked RV dilation (end-diastolic volume of 1,138 ml) with absence of myocardial tissue in the ventricular wall. LV, left ventricle.

We report the case of a young woman with a history of Uhl’s anomaly diagnosed during infancy, with irregular follow-up into adulthood. She later underwent ablation for nodal reentrant tachycardia due to recurrent palpitations. Subsequently, she developed heart failure requiring multiple hospitalizations, and episodes of atrial flutter were documented. Cardiac magnetic resonance (CMR) revealed a markedly enlarged right ventricle (RV) with severely impaired systolic function (end-diastolic volume: 1,138 ml; end-systolic volume: 1,075 ml), extremely thin free walls due to near absence of myocardial tissue, a massively dilated right atrium, and a nondilated left ventricle with moderately reduced systolic function secondary to paradoxical septal motion (Figure 1).

**Figure 2:**
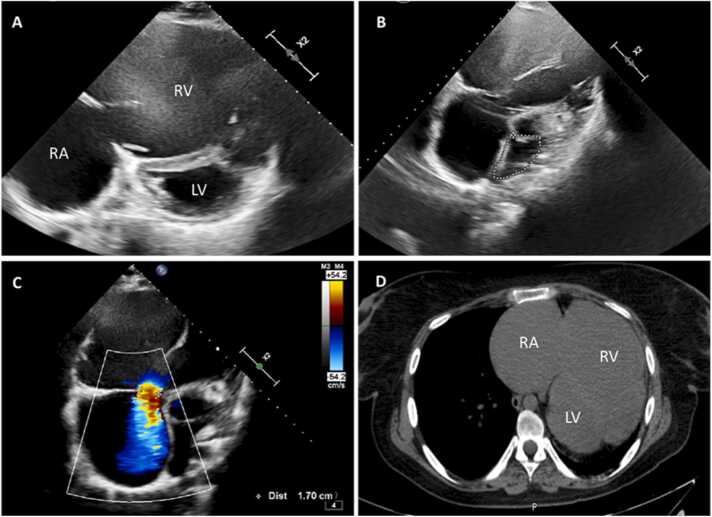
(A) Echocardiography with massive right ventricle (RV) dilation, flattening of the interventricular septum. (B) Compression of the left atrium that reduces its area to 9.9 cm^2^. (C) Severe tricuspid regurgitation due to defective leaflet coaptation, with a vena contracta measuring 15 mm. (D) Chest computed tomography revealed severe right atrium (RA) and RV dilation, associated with leftward mediastinal shift and decreased pulmonary volume.

Upon referral to our center, she presented with New York Heart Association functional class II dyspnea and palpitations. Pretransplant evaluation was initiated. Electrocardiogram showed typical atrial flutter with a variable ventricular response at 75 bpm, confirmed by 24-hour Holter monitoring. Transthoracic echocardiography demonstrated situs solitus with atrioventricular and ventriculoarterial concordance, severe right-sided chamber dilation, a severely hypokinetic and extremely thin-walled RV, and spontaneous echocardiographic contrast in the right cavities. The tricuspid valve was structurally normal but demonstrated severe regurgitation due to poor leaflet coaptation. Paradoxical septal motion and significant left ventricle systolic dysfunction were also noted (Figure 2A-C).

Cardiopulmonary exercise testing revealed a peak VO₂ of 11.5 ml/kg/min (39% of predicted). While flutter ablation was considered, it was deferred due to clinical deterioration and the need for hospitalization.

At the age of 28, she presented with progressive dyspnea, chest pain, and palpitations. Emergency evaluation revealed ventricular tachycardia at 160 bpm accompanied by hemodynamic instability, requiring immediate electrical cardioversion, intravenous amiodarone, and admission to the cardiovascular intensive care unit.

Low-output heart failure ensued, requiring initiation of milrinone, which led to hemodynamic improvement. On physical examination, a positive hepatjugular reflux, hepatomegaly, and a holosystolic murmur at the tricuspid area were noted. Right heart catheterization revealed the following hemodynamic parameters: right atrial pressure 8 mm Hg; pulmonary artery pressure 13/4 mm Hg (mean 10 mm Hg); pulmonary capillary wedge pressure 2 mm Hg; cardiac index of 1.7 liter/min/m², measured by the direct Fick method; systemic vascular resistance 2,000 dyn·s/cm⁵; and pulmonary vascular resistance 232 dyn·s/cm⁵.

Recurrent episodes of nonsustained ventricular tachycardia persisted, leading to her inclusion on the cardiac transplant waiting list.

**Figure 3:**
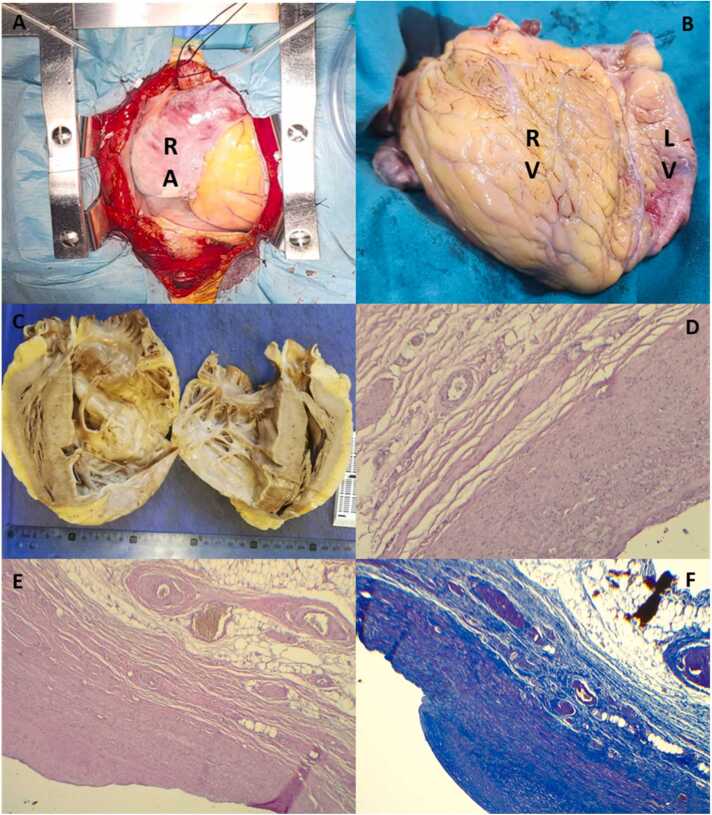
(A) Sternotomy, significant dilation right atrium (RA). (B) Macroscopic image of the explanted heart showing severe dilation of the right ventricle (RV). (C) Macroscopic piece of the explant with massive dilation RV. (D) Photomicrograph of the RV with hematoxylin & eosin (H&E) staining at 10× magnification, showing atrophy of the myocardial layer, adjacent to normal pericardium. (E) Photomicrograph of the RV with H&E staining at 4× magnification, observing myocardial atrophy. (F) Photomicrograph of the RV with Masson's trichrome staining, showing a marked absence of myocardial fibers and subendothelial fibrosis.

Orthotopic heart transplantation was performed using the bicaval technique (Figure 3A and B). Postoperatively, severe mitral regurgitation was noted upon aortic unclamping, attributed to cold ischemia. Conservative management was adopted, with spontaneous improvement to mild regurgitation observed on echocardiography 48 hours later.

The postoperative course was complicated by vasoplegia, hospital-acquired pneumonia, and *Klebsiella pneumoniae* bacteremia.

Gross examination of the explanted heart revealed massive right-sided chamber dilation and marked thinning of the right ventricular free wall, with a maximum thickness of only 0.3 cm (Figure 3C). Histological analysis demonstrated subendocardial fibrosis, extensive myocardial atrophy, and complete absence of myocardial tissue in the right ventricular free wall. Both the endocardium and epicardium preserved their normal histological architecture (Figure 3D-F). These findings confirmed the diagnosis of Uhl’s anomaly and validated the preoperative imaging assessments.

At latest follow-up, she remains asymptomatic under routine surveillance.

## Discussion

We present the case of an adult woman with Uhl’s anomaly successfully treated with orthotopic cardiac transplantation for end-stage heart failure. A review of the literature identified only 2 previously reported cases of heart transplantation in adult patients with this condition. This report represents the third such case, with a favorable clinical outcome, further supporting cardiac transplantation as a viable therapeutic option in adult patients with refractory heart failure due to Uhl’s anomaly.^1^, ^2^

Uhl’s anomaly is an exceptionally rare congenital cardiomyopathy, with an estimated prevalence of fewer than one in a million individuals. It is characterized by partial or complete absence of the right ventricular myocardium. The most widely accepted pathogenic mechanism involves myocardial apoptosis during fetal development. Histopathologically, the hallmark features include a virtually absent myocardial layer in the free wall of the RV, with direct apposition of endocardial and epicardial layers devoid of interposed adipose tissue. In contrast, the interventricular septum, septomarginal trabeculation, and tricuspid valve apparatus are typically preserved.^3^, ^4^

Due to the severity of the anomaly, most affected individuals succumb during infancy or early childhood. In the rare instances of survival into adolescence or adulthood, clinical presentation often includes progressive right heart failure, left ventricular dysfunction related to ventricular interdependence, and both ventricular and supraventricular arrhythmias.^5^

Echocardiography is the first-line tool to detect a dilated, thin-walled, hypokinetic RV and exclude Ebstein’s anomaly. CMR helps differentiate Uhl’s anomaly from arrhythmogenic right ventricular cardiomyopathy by confirming lack of fat infiltration. Right heart catheterization is needed to exclude pulmonary hypertension.^6^, ^7^, ^8^

Gross examination typically reveals significant dilation of the right-sided cardiac chambers, accompanied by marked thinning of the right ventricular free wall, often measuring less than 1 cm in thickness.^10^ Histologically, the condition is characterized by subendocardial fibrosis, extensive myocardial atrophy, and a complete absence of myocardial tissue in the right ventricular free wall. Notably, the endocardium and epicardium are typically preserved.^7^, ^8^

Surgical management of Uhl’s anomaly is not standardized, and data remain limited. In pediatric patients, strategies have included partial or complete right heart exclusion via cavopulmonary anastomoses such as bidirectional Glenn or Fontan procedures. The “one-and-a-half ventricle repair,” which combines right ventricular reduction plasty with a bidirectional Glenn shunt, has also been proposed. In adolescents and adults, surgical options vary depending on the patient’s hemodynamic profile and may include Fontan conversion or procedures aimed at reducing right ventricular volume and improving valve competence, such as free wall plication and tricuspid annuloplasty. However, these approaches are supported only by isolated case reports, and no consensus exists regarding optimal surgical intervention.^9^, ^10^

Our patient exhibited the full clinical spectrum associated with Uhl’s anomaly, including progressive biventricular dysfunction and malignant arrhythmias. This case adds to the limited but growing body of evidence supporting orthotopic heart transplantation as a potentially curative therapeutic strategy in adult patients with Uhl’s anomaly and advanced heart failure. With this evidence, we should stop thinking about palliative surgery and consider heart transplantation as the first option.

## Conclusion

Uhl's anomaly is a rare and often fatal condition with poor prognosis. Survivors into adulthood typically develop progressive heart failure and arrhythmias. Diagnosis depends on echocardiography and CMR. While palliative surgery may relieve symptoms, heart transplantation is the only definitive treatment for end-stage cases.

## Disclosure statement

The authors declare that they have no known competing financial interests or personal relationships that could have appeared to influence the work reported in this paper.

The authors would like to thank Hospital El Cruce for its institutional support and for Provident access to the clinical and imaging resources that made this research possible.

The authors received no financial support for the preparation of this manuscript.

The patient has provided written informed consent for the publication of this case report and any accompanying information or images.

## Data Availability

The data supporting the findings of this study are available from the corresponding author upon reasonable request.
